# Activity of Microbial-Derived Phenolic Acids and Their Conjugates against LPS-Induced Damage in Neuroblastoma Cells and Macrophages

**DOI:** 10.3390/metabo13010108

**Published:** 2023-01-09

**Authors:** Dolores González de Llano, Mikel Roldán, Laura Parro, Begoña Bartolomé, M. Victoria Moreno-Arribas

**Affiliations:** Department of Food Biotechnology and Microbiology, Institute of Food Science Research, CIAL (CSIC-UAM), C/Nicolás Cabrera 9, 28049 Madrid, Spain

**Keywords:** gut phenolic acids, glucoronides, sulfates, neuroprotection, anti-inflammation, oxidative stress, LPS, ROS-scavenging

## Abstract

The aim of this study was to investigate whether microbial-derived phenolic acids, 3,4-dihydroxyphenylacetic (DHPA), protocatechuic acid (PCA), and dihydrocaffeic acid (DHCFA) and their conjugated forms (DHCFA 3-O-sulfate and DHCFA 3-O-β-D-glucuronide), exhibit protective effects against neuroinflammation and oxidative stress. Experiments were performed on human neuronal SH-SY5Y cells stimulated with bacterial lipopolysaccharide (LPS) and *tert*-butyl hydroperoxide (*t*BHP). Anti-inflammatory activity in terms of pro-inflammatory cytokine production was also evaluated in LPS-stimulated RAW 264.7 macrophages as a reactive microglial model. Treatment of the SH-SY5Y cells with the free phenolic acids, as well as with the conjugated metabolites, at physiologically concentrations (1, 10 and 50 μM), resulted in increased cell viability of LPS- and *t*BHP-stimulated cells. Phenolic metabolites and, especially, the conjugated derivatives also protected neuronal cells through significant attenuation of inflammation by decreasing ROS levels. Furthermore, the conjugated and microbial-derived phenolic metabolites significantly inhibited the secretion of proinflammatory cytokines (TNF-α, IL-6, and IL-8) in LPS-stimulated macrophages. Among the phenolic metabolites tested, different efficacies were observed, with the glucuronide form standing out. Overall, these results suggest, for the first time, that conjugated derivatives of phenolic acids seem to be more effective at protecting neurons from inflammation damage and oxidative stress. Further in vivo studies are warranted.

## 1. Introduction

Neurodegeneration represents a major threat to the elderly’s health and is the main pathologic feature of diseases such as Alzheimer’s and Parkinson’s [1,2]. In particular, Alzheimer’s disease (AD) is the major cause of age-related dementia (60–80%), with high morbidity and mortality, for which there is no available cure [2,3]. The etiology and pathogenesis of AD is complex and the mechanisms involved are still not fully understood. Neuroinflammation, one result of immunosenescence, has long been thought to promote progression of AD [4,5]. In addition, oxidative stress processes are considered to mediate the onset of AD [6,7]. Physiologically, AD is mainly characterized by the presence of two aberrant structures in the brain of patients: senile plaques (composed of amyloid-β-peptide, Aβ) and neurofibrillary tangles (whose main component is phosphorylated tau protein) [8,9]. Both events interfere with normal neuronal communication in AD patients, disrupting the signaling process, which are also closely related to neuroinflammation and oxidative damage and other functional losses. 

The “gut microbiota-brain axis” (and, in addition, the “oral and gut microbiota-brain axis) refers to the network of connections involving multiple biological systems that is crucial in maintaining homeostasis of the gastrointestinal, central nervous, and microbial systems of humans [10]. The microbial metabolism plays a prevalent role in these gut-brain communication pathways though “direct” and “indirect” signaling in the regulation of many metabolic, neurochemical, and immunological factors that, from the gut, affect the nervous system [10]. Probably the most known microbial direct signaling is the production of short-chain fatty acids (SCFAs) [11]. Concerning indirect signaling, the microbial metabolism affects the regulation of the neuroendocrine system [12] and the production of neurotransmitters [13]. In relation to the immune system, the bacterial lipopolysaccharide (LPS), that is the major outer surface membrane component present in almost all Gram-negative bacteria, is widely recognized as a potent activator of monocytes/macrophages, affecting the production of inflammatory cytokines and chemokines [14].

Recently, different studies have detected LPS in aged human brain, as well as an increased abundance of it around and within AD-affected neurons [15]. It is inferred that gut microbial-generated LPS would be able to cross the gastrointestinal barriers into the systemic circulation and across the blood–brain barrier (BBB) into the brain [16]. In addition, some evidence indicates the presence of oral anaerobe *Porphyromonas gingivalis* in AD-affected brain and the implication of LPS as a contributor to further neuroinflammation and degeneration [17]. Regarding experimental approaches, LPS administration has been widely used for the investigation of inflammation-associated diseases and testing of anti-inflammatory products in vitro and in vivo [18]. In neuroblastoma SH-SY5Y cells, for example, it was found that LPS treatment resulted in the upregulation of multiple pathways involved in neurological diseases [19]. Microglia, which are the resident macrophages in the brain, are strongly stimulated by LPS, leading to the secretion of pro-inflammatory cytokines, such as TNF-α that further affects neuronal cells [20,21]. Therefore, both SH-SY5Y cells and macrophages stimulated by LPS, either in separate or in co-culture conditions, are employed as models for evaluating neuroprotective activities of pure compounds and complex mixtures [22,23]. 

**Figure 1:**
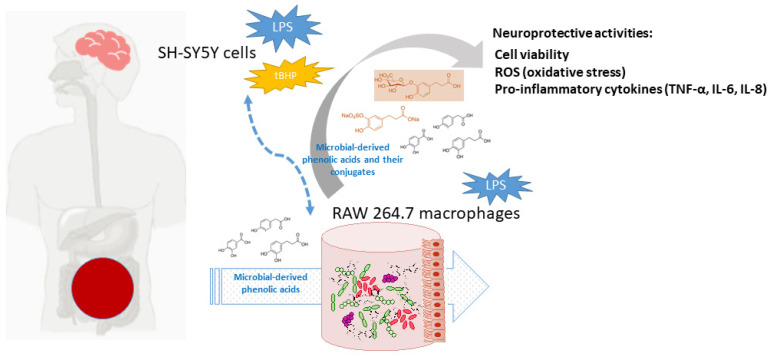
Schematic view of the activity of gut microbiota phenolic metabolites and conjugated forms, and putative protection against neurodegenerative disorders and AD.

Currently, polyphenols are considered dietary components that may protect against neurodegenerative disorders, and AD in particular, through multiple -although quite unraveled- mechanisms [1,5,24]. At the frame of the gut-brain axis, the so-called two-way interaction between polyphenols and gut microbiota seems to be behind the potential neuroprotective action of polyphenols. Dietary polyphenols are mainly metabolized by the gut microbiota, as well as by host enzymes, into a great battery of phenolic acids and other bioavailable metabolites, depending on the polyphenols source (coffee, chocolate, onions, red wine, etc.) and chemical structures, and, in turn, they also appear to modulate the composition and/or metabolic activity of the gut microbiota [25]. Non-digested polyphenols and/or their phenolic metabolites may also act at the host intracellular level, intervening in cell signaling processes related to the integrity of the intestinal barrier, all affecting the functionality of the immune system and regulating inflammatory processes [26]. Low molecular weight metabolites can be partially absorbed and reach targeted organs, where they exert beneficial biological effects [27]. Furthermore, these compounds undergo further biotransformations in the enterocyte and hepatocytes, where they can be conjugated to glucuronides, sulfates, and o-methyl derivatives by the action of phase II enzymes. In fact, these metabolites have been detected in human plasma and physiological fluids at low micromolar concentrations [28]. Several studies in silico, in vitro, and in vivo models have demonstrated an ability of gut-derived metabolites to cross the BBB and reach the brain [29]. In this sense, it has been pointed out that serum-bioavailable metabolites are much more favorable candidates than their corresponding phenolic precursors to overcome cellular barriers and act as effectors for attenuating neuroinflammation [30]. Therefore, the question that remains to be answered is what are the phenolic metabolites that prevents/delays the pathogenesis of neurocognitive disorders after their gut metabolism. Motivated by the scarcity of references to this subject, the aim of this study was to investigate whether some microbial-derived phenolic acids detected in human blood [3,4-dihydroxyphenylacetic (DHPA), protocatechuic acid (PCA), and dihydrocaffeic acid (DHCFA)] and their conjugated forms [dihydrocaffeic acid 3-O-β-D-glucuronide (DHCFAg) and dihydrocaffeic acid 3-O-sulfate (DHCFAs)] exhibit protective effects against neuroinflammation and oxidative stress. Experiments were performed on human neuronal SH-SY5Y cells stimulated with LPS and *tert*-butyl hydroperoxide (*t*BHP), simulating Alzheimer’s disease conditions. Anti-inflammatory activity in terms of pro-inflammatory cytokines production was also evaluated in LPS-stimulated RAW 264.7 macrophages as a reactive microglial model. Figure 1 depicts the target and flow of the research process.

## 2. Materials and Methods

### 2.1. Phenolic Metabolites and Other Chemicals

**Figure 2:**
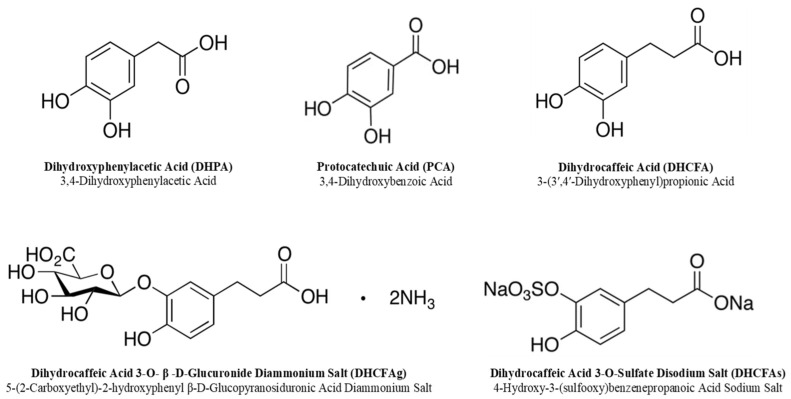
Chemical structures of the phenolic metabolites tested in this study.

**Figure 3:**
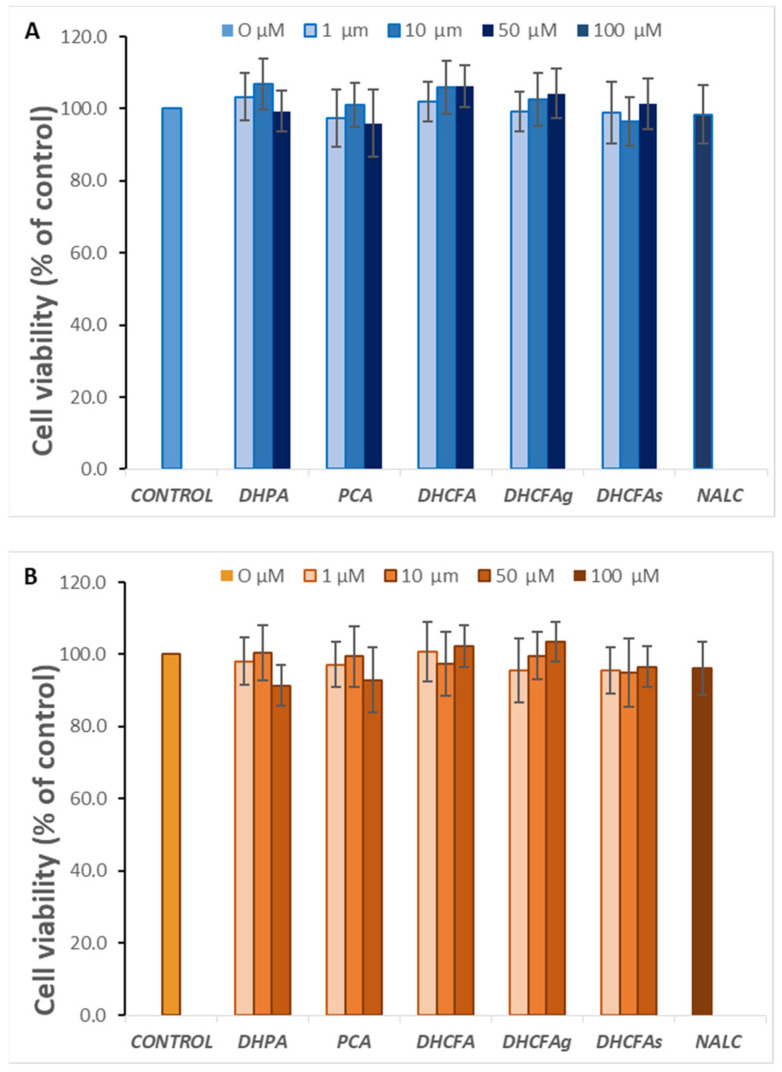
Effect of microbial-derived phenolic acids (DHPA, PCA, and DHCFA) and conjugated derivatives (DHCFAg and DHCFAs), at 1, 10, and 50 μM, on viability (% of the control) of SH-SY5Y cells (**A**) and RAW 264.7 macrophages (**B**), after 24 h of incubation. Data are expressed as mean ± SEM (*n* = 9).

The free and conjugated phenolic acids tested in this study are listed in Figure 2; 3,4-dihydroxyphenylacetic acid (DHPA) and 3,4-dihydroxybenzoic acid (protocatechuic acid; PCA) were purchased from Sigma-Aldrich (San Louis, MO, USA). Dihydrocaffeic acid (3-(3′,4′-dihydroxyphenyl)propionic acid, DHCFA) and its conjugated forms [dihydrocaffeic acid 3-O-β-D-glucuronide (DHCFAg) and dihydrocaffeic acid 3-O-sulfate (DHCFAs)] were purchased from Toronto Research Chemicals, Inc. (TRC, Toronto, QC, Canada). N-acetyl-L-cysteine (NALC), which is a common antioxidant that can directly react with ROS, promote glutathione synthesis, and inhibit nerve cell apoptosis, was obtained from Sigma-Aldrich Chemical Co. (St. Louis, MO, USA) and was used as the antioxidant reference control [31]. Stock standard solutions of these compounds (1 mM) were freshly prepared and filtered (0.22 μm) in sterile Dulbecco’s phosphate buffered saline solution (DPBS, Lonza, Walkersville, MD, USA).

The bacterial lipopolysaccharide (LPS), a pro-inflammatory inducer, and *tert*-butyl hydroperoxide (*t*BHP), used to induce in vitro cellular oxidative stress, were purchased from Sigma-Aldrich (San Louis, MO, USA).

### 2.2. Cell Culture

SH-SY5Y (ATCC^®^ CRL2266™) human neuroblastoma cells (ATCC CRL2266™) and RAW 264.7 murine macrophage cells (ATCC^®®^ TIB-71™) were purchased from the American Type Culture Collection (ATCC). Tissue culture reagents were from Lonza (Slough, UK) and Invitrogen (Paisley, UK). SH-SY5Y neuronal cells were cultured in Dulbecco’s modified Eagle Medium and Ham’s F12 (1:1 *v*/*v*) (Lonza, Slough, UK) supplemented with 10% fetal bovine serum (FBS), 1% antibiotics (100 IU/mL penicillin and 100 μg/mL streptomycin), and 1% non-essential amino acids. Cells were plated at densities of ~1 × 10^6^ cells in 75 cm^2^ culture flasks (Costar; Corning Incorporated, Kennebunk, ME, USA) and incubated at 37 °C under 5% CO_2_ in a humidified incubator until 90% confluence was reached. The sub-culturing cell process was carried out by trypsinization. 

For the maintenance and growth of RAW 264.7 macrophages, cells were cultured in 75 cm^2^ flasks with DMEM high-glucose medium supplemented with antibiotics (1% penicillin/streptomycin) and 10% FBS, and incubated (37 °C and 5% CO_2_), as described above. Cells from confluent monolayers were detached in renew cultured medium using a cell scraper and plated again.

### 2.3. Cytotoxicity Assays

The toxicity against SH-SY5Y and RAW 264.7 cells of the selected phenolic metabolites (Figure 2) was determined by means of cell viability using the colorimetric 3-[4,5-dimethylthiazol-2-yl]-2,5 diphenyl tetrazolium bromide (MTT) assay. Briefly, cells (100 μL/well) were seeded at a density of 4 × 10^5^ cells/mL and 2 × 10^5^ cells/mL for SH-SY5Y and RAW 264.7 cells, respectively, in 96-multiwell plates. Then, they were grown for approximately 24 h to enable cell attachment and to obtain confluent cell monolayers. Afterwards, supernatants were discarded, and monolayers cells were overlaid with the phenolic solutions. These were prepared in culture medium without FBS via serial dilutions from the stock standard solutions (1 mM) at physiological concentrations (1 μM, 10 μM, and 50 μM) based on previous group and literature data [22,32,33]. Then, cell plates were incubated at 37 °C under 5% CO_2_ atmosphere for 24 h. Subsequently, supernatants were replaced by the MTT reagent (0.5 mg/mL) and plates were returned to the incubator (37 °C, 5% CO_2_) for another three hours. Thereafter, supernatants were carefully removed and formazan crystals were dissolved with DMSO and kept away from light. Finally, absorbance was measured at 570 nm using a Cytation 5 multiplate reader, (Biotek, Winooski, VT, USA). Control (no compound added) was considered as the maximum percentage of viability (100%), and the sample values were calculated as the absorbance ratio between the cell culture treated with the phenolic compound and the untreated control × 100 (percentage of control cell viability). Three independent experiments were performed in triplicate. For the maintenance and growth of RAW 264.7 macrophages, cells were cultured in 75 cm^2^ flasks with DMEM high-glucose medium supplemented with antibiotics (1% penicillin/streptomycin) and 10% FBS, and incubated (37 °C and 5% CO_2_), as described above. Cells from confluent monolayers were detached in renew cultured medium using a cell scraper and plated again.

### 2.4. Experiments in SH-SY5Y Cells: LPS-Induced Cell Inflammation and tBHP-Induced Cell Oxidation

For testing the neuroprotective effects of microbial-derived phenolic metabolites in SH-SY5Y cells, they were treated with LPS, to induce inflammation, or with *t*BHP, to induce oxidative stress. In both cases, neuroprotective effects were evaluated by means of cell viability and ROS (reactive oxygen species) accumulation, in separate experiments. For cell viability evaluation, SH-SY5Y cells were cultured and seeded on 96-well plates for 24 h, as described above. The next day, phenolic compounds (1, 10, and 50 µM) and NALC (100 μM) were added to cells, which were simultaneously stimulated by adding LPS (100 ng/mL) or *t*BHP (40 μM) to the medium. Positive controls of cells treated with LPS or *t*BHP, but without phenolic metabolites/NALC, as well as negative controls, which refers to cells without compound treatment or stimulation, were prepared in parallel. In addition, the cells were exposed to phenolic metabolites/NALC without inductors (LPS or *t*BHP) to study their potential protective effects in healthy cells. For all these cases, cell viability after 6 and 24 h of incubation was measured with mitochondrial MTT reagent, as described above. The percentage of cell proliferation values was referred to the control cells (untreated cells and non-induced; 100%). All conditions were performed in triplicate and in three independent experiments.

Similar experiment design was followed for evaluating ROS generation, that was measured using the fluorescent compound 2′,7′-dichlorodihydrofluorescein diacetate (DCFDA) (Sigma-Aldrich; San Louis, MO, USA). Phenolic metabolites (1, 10, and 50 μM) and NALC (100 μM) were added along with the inductor agent (LPS at 100 ng/mL, *t*BHP at 40 μM) to cells, plated in 96-well plates as described above. After 24 h of co-incubation (37 °C; 5% CO_2_) the cell supernatants were removed, the cell monolayers were washed with PBS and the fluorescent reagent (10 μM) was added. Subsequently, the cells were incubated for 2 h at 37 °C and with 5% CO_2_, and kept away from light. The DCF fluorescence was recorded at 488 nm (excitation wavelength) and 525 nm (emission wavelength) using a Cytation 5 reader (Biotek, Winooski, VT, USA). As the control, cells without addition of the compounds and the stimulated agent were used. The fluorescence of ROS species produced in the positive control (with LPS or *t*BHP and without compounds) was established as maximum ROS generation (100%). Quantifications were conducted three-fold in triplicate trials.

### 2.5. Experiments in RAW 264.7 Cells: LPS-Induced Cell Inflammation

Protective effects of microbial-derived phenolic metabolites against LPS-induced damage in RAW 264.7 macrophages were evaluated by means of cell viability, ROS accumulation, and proinflammatory cytokines production, all in separate experiments. For cell viability evaluation and ROS generation, the protocols were the same as described above for the SH-SY5Y cells. Briefly, RAW 264.7 cells were incubated with different concentrations of the phenolic metabolites (1, 10, and 50 µM) and NALC (100 μM), along with LPS (100 ng/mL) for 24 h to induce inflammation. Positive controls of cells treated with LPS, but without phenolic metabolites/NALC, as well as negative controls, which refers to cells without compound treatment or stimulation, were prepared in parallel. In addition, the cells were exposed to phenolic metabolites/NALC without LPS to study their potential protective effects in healthy cells. All cases were assayed after 24 h of incubation in three independent experiments by triplicate.

For evaluating the production of proinflammatory cytokines, the RAW 264.7 macrophages platted in 48-well plates (2 × 10^5^ cells/mL) were incubated for 24 h, as described above. Afterwards, phenolic compounds at physiological concentrations (1 and 10 μM), and NALC (100 μM) were added to the cells, which were either stimulated or not by adding LPS (100 ng/mL) to the medium, and subsequently incubated (37 °C; 5% CO_2_) for 24 h. Then, the cell supernatants were collected, centrifuged, and frozen at −20 °C until use. Levels of proinflammatory cytokines [tumor necrosis factor alpha (TNF-α), interleukin-6 (IL-6), and interleukin-8 (IL-8)] in thawed supernatants were determined by enzyme-linked immunosorbent assay (ELISA) using commercial kits from Thermo Fisher Scientific (Waltham, MA, USA). Cytokines were quantified following the manufacturer’s instructions and by measuring the absorbance at 450 nm using the BioTek Cytation 5 cell imaging multimode reader (BioTek Instruments Inc., Winooski, VT, USA). Levels of TNF-α, IL-6, and Il-8 present in the supernatants from cells treated with phenolic metabolites and activated with LPS were compared to the positive control (LPS stimulated cells but no phenolic metabolites). Results in the absence of LPS were not plotted due to unstimulated cells only releasing slight and comparable levels of proinflammatory cytokines. All the quantifications were performed in triplicate from two independent experiments. (100%). Quantifications were conducted three-fold in triplicate trials.

### 2.6. Statistical Analysis

The results were expressed as mean ± standard error (SEM) of three independent experiments. The t-Student parametric test or Mann Whitney U non-parametric test were applied for related data to evaluate the differences between the control results and the samples, and between the samples from each other. Statistical analysis was carried out using the SPSS program, version 28.

## 3. Results and Discussion

Scientific evidence on the limited absorption and bioavailability of polyphenols and their extensive metabolism by human gut microbiota [24,29,34] have raised some concerns about the physiological relevance of previous experiments, in which the activity of parenteral phenolic compounds on neuronal function was tested at supra-physiological concentrations, and considering unabsorbed prototypes. Therefore, the selection of low molecular weight phenolic metabolites generated by the intestinal microbiota and their glucuronide and sulfate conjugated derivatives, occurring in plasma, was carried out based on previous studies suggesting their availability to cross the blood–brain barrier [29,30,32,35]. 

The three microbial-derived phenolic acids selected (DHPA, PCA, and DHCFA) hold the 3,4-dihydroxy substitution in the benzene ring, only differing in the hydrocarbon side chain. Regarding DHPA, this is a known microbial metabolite derived from quercetin, among other polyphenols, and has shown antioxidant and anti-inflammatory effects on neuronal function in previous studies [32,33]. PCA is a main metabolite of the anthocyanin cyanidin 3-glucoside present in red wine, as well as in berries, and it has been found that nearly of 30% of orally administered PCA can be recovered in the rat brain [36]. DHCFA is a metabolite of caffeic acid with potent antioxidant properties [37]. On the other hand, the main conjugated forms of DHCFA, the 3-O-β-D-glucuronide (DHCFAg) and the 3-O-sulfate (DHCFAs), were assayed to shed light on the hypothesis of whether conjugated derivatives (glucuronides and sulfates) can improve the efficacy of their precursors, in preventing inflammation and oxidative stress prevalent in neurodegenerative disorders [22,29,35].

### 3.1. Cytotoxicity of Microbial-Derived Phenolic Acids and Their Conjugates

With regard to a possible cytotoxic effect of the tested compounds on SH-SY5Y human neuroblastoma cells, none of the compounds altered cell viability at the selected physiological concentrations (1, 10, and 50 µM) after 6 h (data not shown) or 24 h of incubation (Figure 3A). Similarly, none of these metabolites were cytotoxic for RAW 264.7 macrophages at any of the concentrations tested (Figure 3B). 

### 3.2. Activity of Microbial-Derived Phenolic Acids and Their Conjugates in the SH-SY5Y Neuronal Model

#### 3.2.1. Activity under LPS-Induced Cell Inflammation: Cell Viability and ROS Accumulation 

Inflammation is an essential physiological process against injury, pathogens, as well as other harmful inducements, but enhanced and prolonged inflammation causes dysfunction and inflammatory diseases, as well as neurodegeneration which triggers dementia, AD. As none of the doses of the compounds selected showed cytotoxic effects, we proceeded to test which of them had a greater effect on reducing LPS-induced neuroinflammation. Therefore, the potential neuroprotective effect of microbial phenolic metabolites (DHPA, PCA, and DHCFA) and conjugated derivatives (DHCFAg and DHCFAs), at 1, 10, and 50 μM on SH-SY5Y cell viability against induced inflammation were evaluated after 6 and 24 h of co-incubation with LPS.

**Figure 4:**
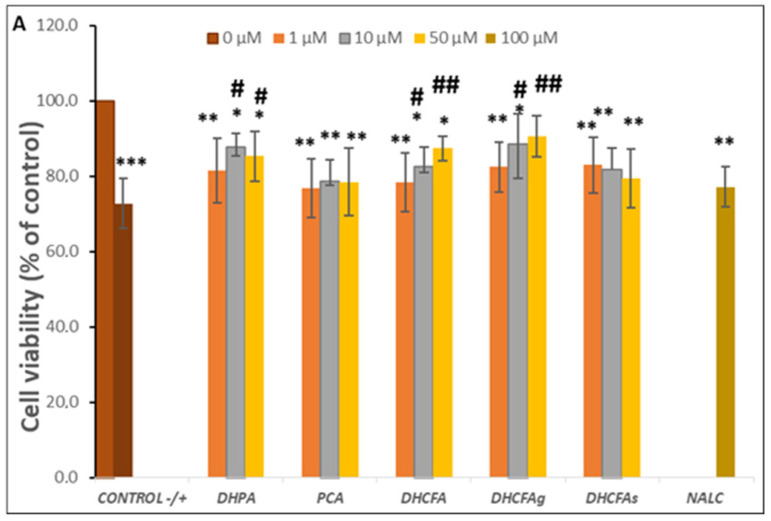

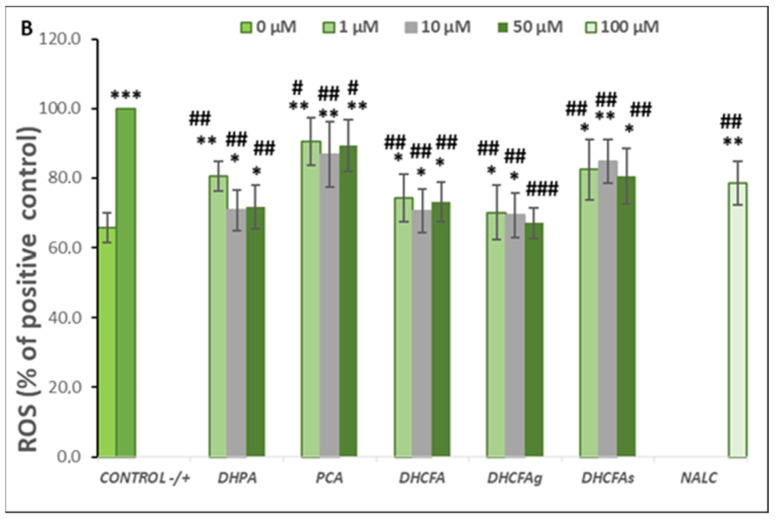
Neuroprotective effects of microbial-derived phenolic acids (DHPA, PCA, and DHCFA) and conjugated derivatives (DHCFAg and DHCFAs) (1, 10, and 50 μM) on SH-SY5Y cells under LPS (100 ng/mL) inflammation damage for 24 h of co-incubation. Results of cell viability (% of the control) (**A**) and ROS generation (% of the positive control) (**B**) in LPS-stimulated cells. *, **, *** Significant differences (*p* < 0.05, *p* < 0.01, *p* < 0.001, respectively) with respect to the control (−); ^#^, ^##^, ^###^ Significant differences (*p* < 0.05, *p* < 0.01, *p* < 0.001, respectively) relative to the positive control (+).

The viability of SH-SY5Y cells after 24 h of LPS stimulation (positive control) significantly decreased by up to 30% with respect to the untreated cells (without compounds or LPS, negative control) (*p* < 0.001, ***) (Figure 4A). However, the co-treatment with all phenolic metabolites for 24 h decreased the LPS-induced cytotoxicity compared to the positive control (72.75 ± 5.70). Significant differences (*p* < 0.05, ^#^; *p* < 0.01, ^##^) were observed after incubation with the metabolites DHPA and DHCFA, as well as its glucuronide DHCFAg, at 10 and 50 µM concentrations, which protected neuronal cells against LPS-inflammation damage (Δ cell viability ≥ 20%) (Figure 4A). In this lane, some phenylacetic acids and phenylpropionic acids had also showed neuroprotective effects and modulation of neuroinflammatory pathways in the same neuronal cell model stimulated with the SIN-1 nitrosative stress by significantly decreasing ERK 1/2 activation and mitogen-activated protein kinase (MAPK) p38 [32]. The most noticeable reduction of LPS-induced cytotoxicity was found in the cells incubated with the conjugated metabolite, DHCFAg at 50 µM, which nearly restored the viability values (~90%) of control. However, treatment with the sulfate conjugated metabolite (DHCFAs) only led to slight non-significant effects (Figure 4A). These results showed conjugated selectivity of the DHCFA metabolite, in which the glucuronides exhibited noticeable neuroprotective activity, while its sulfate form exhibited no obvious protective effect, in concordance with previous studies [22]. A similar trend was observed after PCA treatment, with non-significant differences (*p* > 0.05) characterized by a small increase in viability compared to untreated cells (Figure 4A). Conversely, beneficial effects of catechol and its conjugated sulfates [38], as well as of PCA [39], that decreased LPS-induced damage in the BV-2 microglia cell line, were previously reported. Regarding the reference antioxidant, NALC also had non-significant (*p* > 0.05) effects against LPS–induced inflammation and hardly reduced its cytotoxicity, in contrast to its ability in significantly lessening *t*BHP-induced oxidative stress, as described below.

A similar trend in cell viability, although to a lesser extent, was observed when neuronal cells were treated with LPS for 6 h (results not shown). Cell viability under LPS-induced inflammation decreased to 81.58 ± 5.34 with respect to the negative control (untreated cells), and significant changes (*p* < 0.01, **) were also observed in the viability of cells co-incubated with conjugated metabolites. However, slightly protective effects compared to the positive control (cells without phenolics but with LPS) were observed after 6 h of incubation with DHPA (10 µM), DHCFA, and its glucuronide (50 µM) (cell viability ≥ 10%) (results not shown). Therefore, the protective effects of free and conjugated phenolic acids against LPS-induced damage in SH-SY5Y cells appeared to increase with metabolite exposure time, suggesting a continued supply of metabolites for protection maintenance.

Reactive oxygen species (ROS) are involved in inflammatory disorders and immunomodulation, and are known to be a key factor in neuronal cell death, leading to neurodegeneration and cognitive decline. ROS scavenging effects of the microbial-derived phenolic metabolites (DHPA, PCA, and DHCFA), and conjugated derivatives (DHCFAg and DHCFAs), at 1, 10, and 50 μM on SH-SY5Y cells under LPS-induced inflammation were measured after 24 h of co-incubation (Figure 4B). As expected, LPS treatment resulted in a significant increase (*p* < 0.001, ***) in ROS concentration with respect to the untreated cells (65.71 ± 4.35% with respect to the positive control) (Figure 4B). Notably, this increase in ROS generation after LPS treatment was attenuated in the presence of all the compounds tested (Figure 4B). In particular, after 24-h co-treatment with DHPA, a significant (*p* < 0.01, ^##^) attenuation (~30%) of the high level of ROS of the positive control (100%) was found, standing out at 10 and 50 μM DHFA (Figure 4B). Similarly, González-Sarrías et al. [33] reported that 3,4-dihydroxyphenylpropionic acid, 3,4-dihydroxyphenylacetic acid, gallic acid, ellagic acid, and urolithins prevented neuronal LPS-induced damage via attenuation of ROS levels.

DHCFA also had a higher protective effect under these experimental conditions and significantly (*p* < 0.01, ^##^) inhibited ROS generation in LPS-induced cells (Figure 4B). Nevertheless, the glucuronide DHCFAg metabolite, was the most effective, in a dose-dependent manner, protecting neuronal cells against LPS-inflammation by regulating cell proliferation and ROS-scavenging (*p* < 0.001, ^###^), which, at 50 μM, reached non-significant differences compared to unstimulated cells (control; (*p* > 0.05). However, the other derivative, the sulfate DHCFAs produced less attenuation of ROS levels, in concordance with previous findings [22]. Furthermore, PCA hardly showed any protective effects on restoring cellular antioxidant enzyme activity and cell viability against LPS-induced inflammation (Figure 4B), as seen above for cell viability. However, other studies have reported that PCA had potent anti-inflammatory effects in BV-2 LPS-stimulated microglia cells [39]. PCA also shows a potential role in inhibiting aggregation and fibril destabilization of Aβ and αS proteins [40]. Recently, Song et al. [41] reviewed the pharmacological activities of PCA, including its antioxidant, anti-inflammatory, and neuroprotective effects, among others, suggesting that PCA displays a dual-way modulatory effect on some activities. This is because PCA can inhibit cell survival and proliferation at high concentrations, but its absorption and elimination rate are faster, with glucuronidation and sulfation being the major metabolic pathways [41].

#### 3.2.2. Activity under tBHP-Induced Cell Oxidation: Cell Viability and ROS Accumulation

On the other hand, we undertook the in vitro study of the molecular mechanisms associated with the neuroprotective and anti-inflammatory effects of phenolics in SH-SY5Y cells, including the induction of oxidative stress as part of the neuroinflammation. Oxidative stress, as an imbalance between ROS production and antioxidant defense, is implicated in the etiology of neurodegenerative diseases. In in vitro assays, *t*BHP is commonly used as the ROS inducer [42]. Thus, SH-SY5Y cells treated with *t*BHP are considered models of oxidative stress damage in neuronal cells [31,43]. 

**Figure 5:**
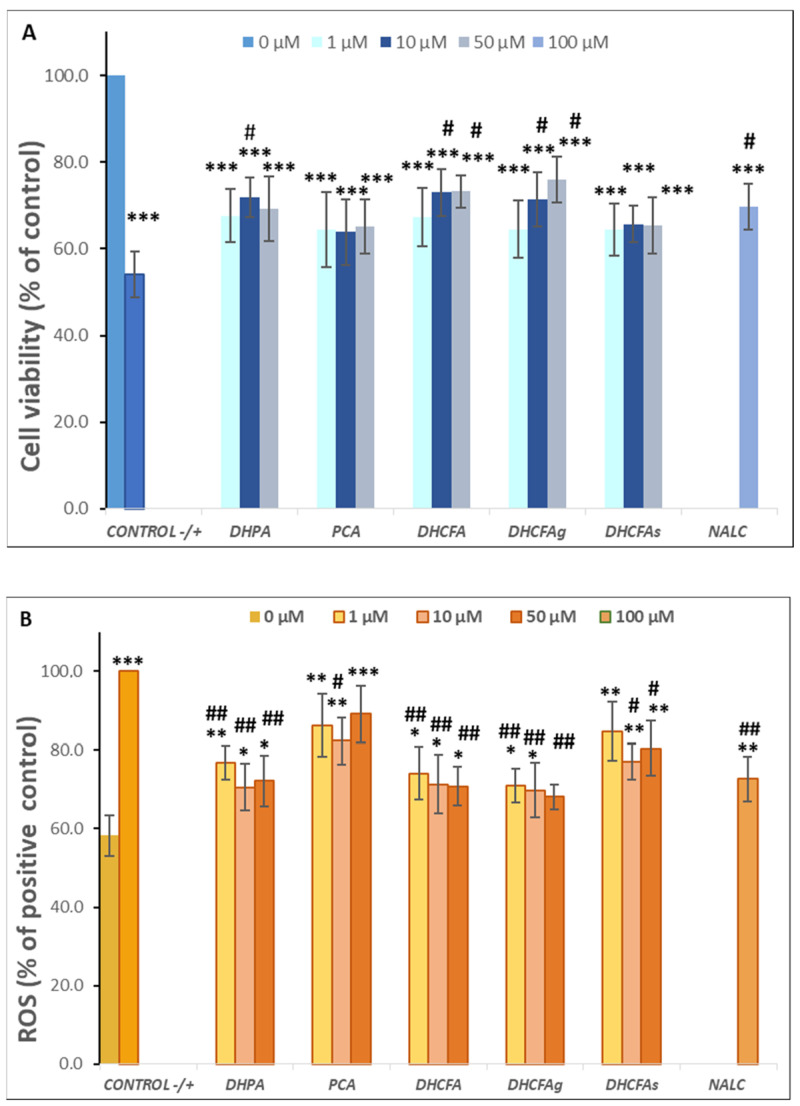
Neuroprotective effects of microbial-derived phenolic acids (DHPA, PCA, and DHCFA) and conjugated derivatives (DHCFAg and DHCFAs) (1, 10, and 50 μM) on SH-SY5Y cells under *t*BHP (40 μM)-induced cell oxidation damage for 24 h of co-incubation. Results of cell viability (% of the control) (**A**) and ROS generation (% of the positive control) (**B**) in *t*BHP-stimulated cells. *, **, *** Significant differences (*p* < 0.05, *p* < 0.01, *p* < 0.001, respectively) with respect to the control (−); ^#^, ^##^ Significant differences (*p* < 0.05, *p* < 0.01, respectively) relative to the positive control (+).

Regarding the neuroprotective effects on these neuronal cells after 24 h of incubation with metabolites and stimulated with *t*BHP (40 μM), the results showed that the *t*BHP-induced oxidation process caused a significant decrease on SH-SY5Y cell viability compared to the control (cells without compounds or *t*BHP) (*p* < 0.001, ***) (Figure 5A). Cell death was around 45-50% in the positive control and was similar to data reported from analogous studies [31,43,44]. On the other hand, the 24-h co-treatment with the tested metabolites protected the neuronal cells from oxidative damage and cell death, even detecting cell viability increases of more than 25% with respect to the positive control with DHPA, DHCFA, and its glucuronide derivative (Figure 5A). In relation to DHPA (3,4-dihydroxyphenylacetic acid), no dose-dependent effect was observed, as the 10 µM concentration was the most effective against oxidative damage (Δ cell viability ≥ 30%), but both (10 and 50 µM) significantly (*p* < 0.05,·^#^) increased SH-SY5Y cell viability (Figure 5A). Similarly, in a previous study by our group using the same neuronal cell line in an inflammatory process induced by the prooxidant SIN-1 and similar doses of some of the tested metabolites, we also found that a pretreatment with 10 µM DHPA produced an increase in cell viability without a dose-dependent effect [32]. In the same way, Cheng et al. [43] also reported that treatment with the chloroform and aqueous fractions from nonis juice significantly weakened the *t*BHP-induced cytotoxicity, using the same *t*BHP concentration (40 µM) and the SH-SY5Y cell line. These authors described that this may be due to the antioxidant and neuroprotection potential for the high total phenolic and flavonoid content of noni extracts. In another study, SH-SY5Y neuronal cell death induced with H_2_O_2_ (300 μM) halved cell viability relative to untreated cells (48.60 ± 0.93%); however, treatment with three flavonoids from *Acer okamotoanum* at 10 μg/mL increased cell viability to 61–70% [44].

Notably, co-treatment of cells with DHCFA and its glucuronide form (DHCFAg) showed the greatest effects on improving the survival of SH-SY5Y cells against oxidative damage by tBHP, and it was possible to establish a significant dose-dependent relationship (Figure 5A). The 50 µM concentration of both DCHF compounds significantly increased cell viability (35%) compared to the positive control (*p* < 0.05, ^#^). In contrast, non-significant effects on the protection against tBHP damage caused by the sulphate derivative (DHCFAs) were observed with respect to the positive control (*p* > 0.05) at any concentration. These results were similar to those reported by Peñalver et al. [22], who also described that only the glucuronides of all tested metabolites derived from resveratrol and its stilbene analog, pterostilbene, exerted a neuroprotective effect on SH-SY5H cells in an H_2_O_2_ oxidation model after pre- and co-treatment (6 and 24 h) with phenolic metabolites. While the rest of the compounds, including the sulphated forms, did not show neuroprotection at the highest concentration tested. Furthermore, a significant neurotrophic effect (*p* < 0.05, ^#^) of the reference antioxidant, NALC, against tBHP-induced oxidative stress was observed, as expected, and was in agreement with previous studies [31,43]. 

As seen from the LPS-induced inflammation experiments, the results of SH-SY5Y cell viability after 6 h of incubation indicated that the treatment with the pro-oxidant agent (*t*BHP) significantly (*p* < 0.01) decreased neuronal cells viability (74.23 ± 5.19%) compared to the negative control (results not shown), but at to a lower extent than that observed for 24 h (Figure 5A). Furthermore, co-incubation with the tested phenolic metabolites did not produce significant changes in cell viability after 6 h with respect to the *t*BHP-positive control, nor were any protective effects against *t*BHP oxidative damage found (Δ cell viability ≤ 10%) (results not shown). As previously mentioned for the LPS-induced model, protective effects of free and conjugated phenolic acids against *t*BHP-induced damage in SH-SY5Y cells became more evident with longer incubation times.

In order to evaluate the protective effects of the phenolic metabolites against cellular oxidative stress in the SH-SY5Y cells, ROS accumulation was measured by the DCFH-DA method (Figure 5B). As expected, a significant increase (*p* < 0.001, ***) in intracellular ROS concentration after cells stimulated for 24 h with *t*BHP (40 μM) was found (Figure 5B). The results demonstrated that the high ROS level of the positive control (100%) was significantly attenuated (*p* < 0.01, ^##^) by incubating for 24 h with the metabolites DHPA, DHCFA, and its glucuronide derivative (Figure 5B). The 24-h co-treatment with DHFA could be suppressed by around 25–30% of ROS generation, relative to that of the positive control, with the greatest effect observed at 10 μM (70.55 ± 6.01), as found in the cell viability. Kim et al. [44] also suggested that three flavonoids from *A. okamotoanum* significantly attenuated ROS-scavenging and protected against cellular oxidative stress, possibly through regulating inflammation and apoptosis.

A noticeable reduction in intracellular reactive oxygen was observed for DHCFA and its glucuronide derivative, DHCFAg, which significantly attenuated ROS levels (*p* < 0.01, ^##^) in a dose-dependent manner, reaching values close to ROS values of unstimulated (control) cells at 50 mΜ (Figure 5B). Once more, both metabolites were highly effective in protecting neuronal cells against cellular oxidative stress, possibly through regulation of inflammation, cell proliferation, and ROS-scavenging. However, the sulfate DHCFA derivative had less ROS scavenging potential, which is in agreement with previous studies that reported only positive effects for glucuronides [22]. In our study, the PCA metabolite showed hardly any protective effects in restoring cellular antioxidant enzyme activity or cell viability, as also seen in the LPS-induced cell inflammation model. 

### 3.3. Activity of Microbial Phenolic Acids and Their Conjugates in RAW 264.7 Macrophage Cells

#### Activity under LPS-Induced Cell Inflammation: Cell Viability, ROS Accumulation, and Cytokines Production

**Figure 6:**
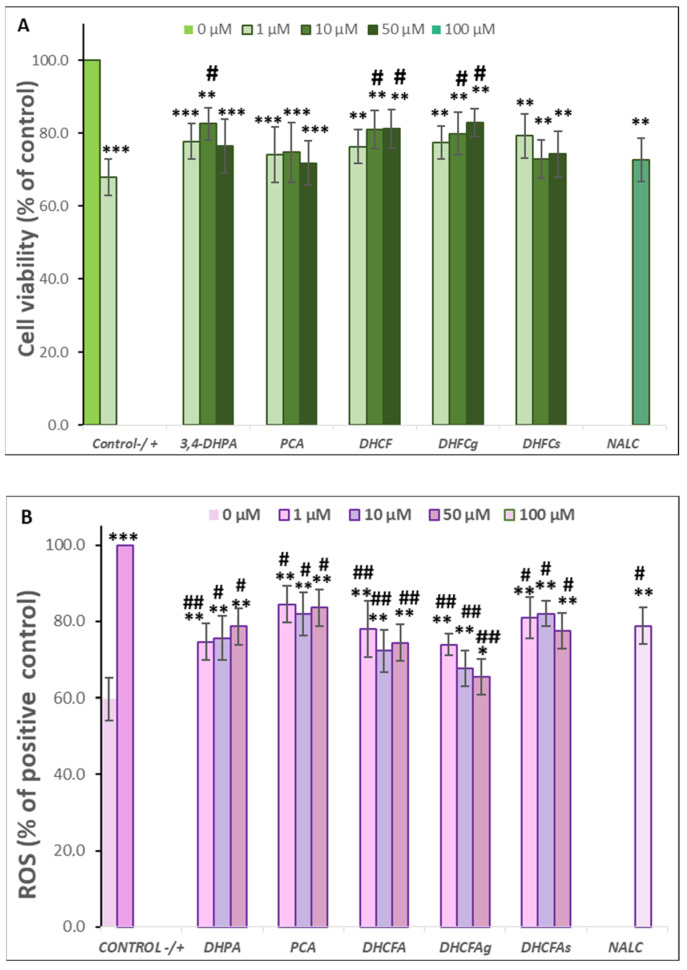
Protective effects of microbial-derived phenolic acids (DHPA, PCA, and DHCFA) and conjugated derivatives (DHCFAg and DHCFAs) (1, 10, and 50 μM) on RAW 264.7 macrophages under LPS (100 ng/mL) inflammation damage for 24 h of co-incubation. Results of cell viability (% of the control) (**A**) and ROS generation (% of the positive control) (**B**) in LPS-stimulated cells. *, **, *** Significant differences (*p* < 0.05, *p* < 0.01, *p* < 0.001, respectively) with respect to the control (−); ^#^, ^##^ Significant differences (*p* < 0.05, *p* < 0.01, respectively) relative to the positive control (+).

**Figure 7:**
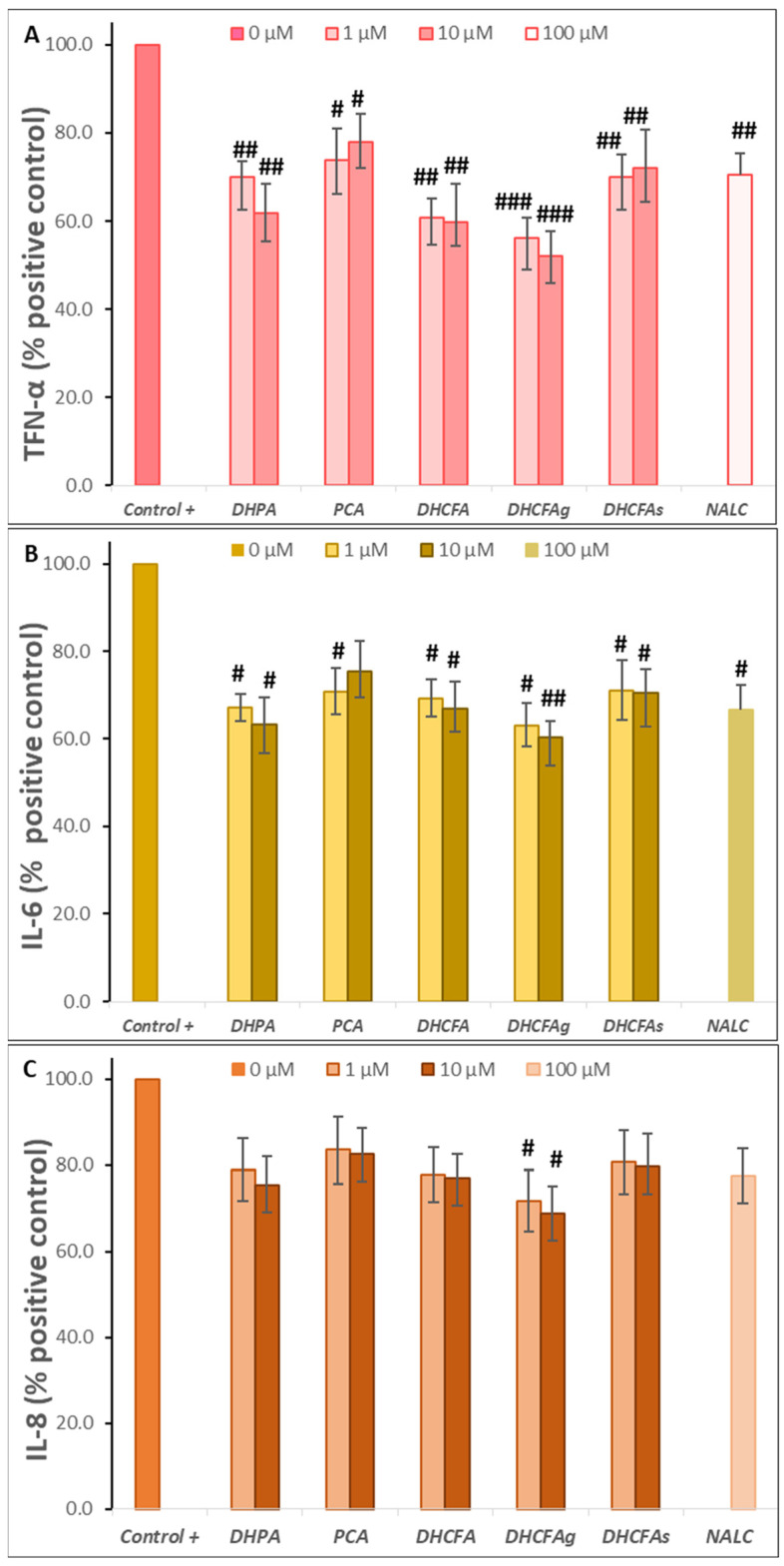
Effects of microbial-derived phenolic acids (DHPA, PCA, and DHCFA) and conjugated derivatives (DHCFAg and DHCFAs) (1 and 10 μM) on the production of proinflammatory makers in RAW 264.7 macrophages under LPS (100 ng/mL) inflammation damage for 24 h of co-incubation: (**A**) TNF-α, (**B**) IL-6, and (**C**) IL-8. ^#^, ^##^, ^###^ Significant differences (*p* < 0.05, *p* < 0.01, *p* < 0.01, respectively) relative to the positive control (+).

Macrophages are noticeable innate immune effector cells, which respond to inflammation and immune modulations [45,46]. When they are activated, exogenous inflammatory inductors (e.g., LPS) stimulate immune responses by inducing the MAPK and NF-B signaling pathways, which up-down the expression of pro-inflammatory cytokines (TNF-, IL-6 and IL-1). Thus, the anti-inflammatory capacity of phenolic acids and their conjugates in RAW 264.7 macrophages under LPS-induced cell inflammation were evaluated by measuring cell viability and ROS, and cytokines production (Figure 6 and Figure 7).

Regarding their effects on RAW 264.7 macrophage viability, co-treatment with the metabolites ameliorated the high LPS-induced cytotoxicity in macrophages, increasing their cell survival (Δ cell viability ≥ 20%) compared to the LPS-induced control, in which its viability decreased to 67.84 ± 4.95 (Figure 6A). In the same way as in neuronal cells, a significant increase (*p* < 0.05, ^#^) was found with the metabolite DHFA (10 µM), and the DHCFA, and its glucuronide DHCFAg (10 and 50 µM) after 24 h of incubation, compared to the positive control (LPS-induced without phenolics). However, less protective effects than the viability of SH-SY5Y neuronal cells under the LPS-induced inflammation were found, probably due to the higher LPS-cytotoxicity in macrophages. Similarly, the conjugated DHCFAs also showed fewer effects on macrophage viability, in concordance with previous findings in similar cells that reported only positive effects for glucoronides [22]. Moreover, the other tested metabolites and the reference antioxidant NALC, also showed non-significant (*p* > 0.05) protective effects preventing macrophages death activated by LPS treatment.

Figure 6B displays the effects of microbial-derived phenolic acids and their conjugates on ROS generation in LPS-stimulated RAW 264.7 macrophages for 24 h of co-incubation. A higher level of intracellular ROS production was observed in LPS-stimulated macrophages (positive control) compared to the untreated cells (control) (59.71 ± 5.48% of the positive control). Significant decreases in ROS concentrations with respect to the positive control (*p* < 0.01, ^##^) were found after 24-h incubation of LPS-stimulated macrophages with the metabolites DHPA at 1 μM, as well as DHCFA and its glucuronide DHCFAg at all concentrations tested (1, 10, and 50 μM), showing an intensely anti-inflammatory potential. Again, the glucuronide DHCFAg showed a strong ROS scavenging potential (35% at 50 μM), whereas the sulfate derivative DHCFAs hardly showed any noticeable ROS reduction (nearby 20%), in concordance with previous findings in similar conditions [22]. The 24-h co-treatment with PCA and NALC also had a lower ROS scavenging potential and significantly (*p* < 0.05, ^#^) suppressed ROS generation (~16–22%) in LPS-stimulated macrophages relative to the positive control. Although it has been reported that PCA could reduce oxidative stress and tissue damage by reducing ROS and other inflammatory markers, the anti-inflammatory potential and antioxidant properties of PCA are unclear and lack reliable clinical support [41]. 

In addition, the potential anti-inflammatory activity of the phenolic metabolites at physiological concentrations was assessed by quantifying the release of proinflammatory cytokines [tumor necrosis factor alpha (TNF-α), interleukin-6 (IL-6) and interleukin-8 (IL-8)] under LPS-induced inflammation. TNF-α plays a crucial role in cell survival, apoptosis, and other cellular processes through regulating immune responses and inflammation by activation of NF-κB and MAPKs signal pathways. Meanwhile, IL-6 expression is associated with inflammation and autoimmunity, and its dysregulation can lead to anomalous immunity and chronic inflammation [47].

As expected, the LPS non-stimulated RAW 264.7 macrophages produced a low steady background level of the three cytokines (<140 pg/mL) (results not shown). However, after 24-h LPS stimulation, the TNF-α, IL-6, and IL-8 production by macrophages significantly increased (*p* < 0.001, ***) reaching values of 406.53 ± 34.49 pg/mL, 314.29 ± 36.27 pg/mL, and 828.36 ± 51.57 pg/mL, respectively (results not shown). Previous studies reported that LPS-activated RAW 264.7 cells increased the production of pro-inflammatory cytokines (e.g., TNF-α, IL-1, and IL-6) and the up-regulated expression of inflammatory mediators (e.g., iNOS and COX-2) [22,46]. Communication imbalances between the brain and the immune system occur in neurodegenerative disorders, that generate a strong release of pro-inflammatory cytokines, such as the interleukins IL-6, IL-1β, TNF-α, and interferon (IFN)-γ [48]. 

As shown in Figure 7A–C, the presence of the tested phenolic metabolites (1 and 10 μM) strongly attenuated the production of pro-inflammatory cytokines derived from the LPS stimulation in LPS-activated RAW264.7 cells. A significant reduction (*p* < 0.05, ^#^; *p* < 0.01, ^##^) of TNF-α and IL-6 values (~40 and 50%, respectively) was found compared to cells stimulated with LPS alone, making the effect on IL-8 production less relevant. Conversely, PCA treatment hardly inhibited the expression of pro-inflammatory molecules after inducing cellular inflammation with LPS. In contrast, Wang et al. [39] reported that protocatechuic acid modulated LPS-induced inflammation in BV-2 microglia cells by inhibiting the production of TNF-α, IL-6, IL-1β, and PGE2 and down-regulating TLR4 expression, as well as suppressing the activation of NF-κB and MAPKs.

Among the phenolic acids tested, DHPA and DHFC strongly blocked the release of all three pro-inflammatory molecules, and especially TNF-α and IL-6 (Figure 7). However, PCA treatment hardly inhibited the expression of pro-inflammatory molecules after inducing cellular inflammation with LPS. In contrast, Wang et al. [39] reported that protocatechuic acid modulated LPS-induced inflammation in BV-2 microglia cells by inhibiting the production of TNF-α, IL-6, IL-1β, and PGE2 and down-regulating TLR4 expression, as well as suppressing the activation of NF-κB and MAPKs. Notably, the glucuronate metabolite (DHFCg) showed the highest anti-inflammatory activity, decreasing the three cytokines production, and especially TNF-α (*p* < 0. 001, ^###^; almost 50% of the positive control), although it is also significant for IL-6 (*p* < 0.01, ^##^); 60.14% of the positive control at 10 µM) and IL-8 (*p* < 0.05, ^#^) (Figure 7). NALC also moderately decreases the secretion of these cytokines, thus demonstrating its effect as a positive anti-inflammatory control, as previously described [31]. In this line, 3-methyl-4′-glucuronide-resveratrol has been described to ameliorate LPS-mediated inflammation in macrophages via inhibition of IL-6 and nitric oxide production, but this IL-6 inhibition was not found for sulfate derivatives of resveratrol [22]. Yang et al. [49] also described that the alkaloid, hygenamine, present in plants, significantly inhibits the production of TNF-α and interleukin 6 (IL-6), as well as ROS in LPS-activated BV2 microglia cells. 

Thus, from this test it can be concluded that some of the phenolic metabolites tested strongly suppressed the production of pro-inflammatory mediators in LPS-stimulated RAW264.7. DHFCg has the highest/greatest capacity to inhibit the secretion of pro-inflammatory cytokines at the physiological concentrations used (1 and 10 µM). However, further studies are needed to confirm this promising anti-inflammatory activity. Accumulating evidence highlights the ability of flavonoids to inhibit the production of pro-inflammatory cytokines, such as TNF-α, IL-6, and IL-1, in neuronal and microglial cells, suggesting close involvement in pathways such as NF-κB or MAPK [50,51]. Blueberry extract polyphenols inhibited the production of IL-1β and TNF-α in LPS-activated microglial cells [52]. Recently, Xagorari et al. [53] reported that the flavonoid luteolin inhibits the expression of LPS-induced proinflammatory molecules due to its interference with LPS inflammatory signaling pathways by reducing the activation of several MAPK family members, and that its inhibitory action on TNF-α release correlates with the inhibition of ERK, p38, and CK2 activation. Kong et al. [46] reported that 5-hydroxymethylfurfural, found in many food products, such as honey, dried fruits, coffee, and black garlic extracts, could exert anti-inflammatory activity in the LPS-induced inflammatory response by inhibiting cytokines, as compared with the non-LPS-stimulated cells.

In summary, the results regarding the activity of in vivo relevant phenolic acids and their conjugates at physiological concentrations (1, 10, and 50 µM) were consistent among the different cell models (SH-SY5Y human neuroblastoma cells and RAW 264.7 murine macrophages), physiological conditions (LPS-induced inflammation and *t*BHP-induced oxidation), and targeted effects (cell viability, ROS accumulation, and proinflammatory cytokine production) explored in this study. In relation to cytoprotective effects (cell viability), the phenolic acids DHPA and DHCFA, and the glucuronide derivative (DHCFAg), led to statistically significant (*p* < 0.05 or *p* < 0.01 with respect to the positive control) protection (higher cell viability) improvement in both cell models and both physiological conditions, whereas no significant (*p* > 0.05) protection was observed for PCA or the sulfate derivative (DHCFAs). ROS accumulation, as a parameter associated with both oxidative stress and inflammation, was significantly (*p* < 0.05, *p* < 0.01, or *p* < 0.01) attenuated in the presence of all the metabolites under both LPS-induced inflammation (SH-SY5Y cells and RAW 264.7 macrophages) and *t*BHP-induced oxidation (SH-SY5Y cells) conditions. For the macrophages model, co-incubation of LPS with the phenolic metabolites also inhibited the production of pro-inflammatory cytokines (i.e., TNF-α), being the more effective (*p* < 0.001) glucuronide derivative (DHCFAg) than the free acid (DHCFA) and its corresponding sulfate derivative (DHCFAs) (*p* < 0.01), as well as the other free acids, DHPA (*p* < 0.01) and PCA (*p* < 0.05). Therefore, the chemical structure of phenolic metabolites in terms of hydrocarbon side chain (DHCFA vs. DHPA and PCA) as well as conjugation (glucuronide vs. sulfate) seemed to condition neuroprotective activity, under the models explored in this study. On the other hand, N-acetyl-L-cysteine (NALC) (100 µM), used as the reference compound, significantly (*p* < 0.05, *p* < 0.01) attenuated ROS accumulation, but no significant (*p* > 0.05) improvement was observed in cell viability for both LPS-induced inflammation models (neuronal cells and macrophages), confirming its main potential as an antioxidant. 

## 4. Conclusions

Gut microbial metabolism is essential in the communication pathways of the “gut microbiota-brain axis”, a bidirectional system maintaining body homeostasis and normal central nervous functionality that has been linked to healthy gut function. Interplays between polyphenols and gut microbiota seem to be behind the potential neuroprotective action of polyphenols, which has been lately associated with bioaccessible low molecular metabolites. However, only a few physiologically relevant studies have focused on the circulating phenolic metabolites and their conjugated forms that might overcome the BBB and reach brain tissues. Moreover, little research involving these conjugated metabolites, glucuronides, and sulfates, has been conducted in experimental models of AD/neurodegeneration and the mechanisms that might account for their neuroprotective action. In this work, in vitro neuroprotective and anti-inflammatory effects, as well as the plausible molecular mechanisms associated with them, were evaluated in two different models of neurodegeneration: SH-SY5Y cells as models of neuronal cells, and RAW 264.7 macrophages as a reactive microglial model. Experiments were performed under LPS-induced inflammation and *t*BHP-induced oxidative stress, two physiological conditions that are involved in Alzheimer’s disease and neurodegeneration disorders. 

The results provide compelling evidence that gut phenolic metabolites, and, in particular, conjugated glucuronides interact with neuronal cell functioning and are able to reduce LPS- or *t*BHP-induced damage by enhancing neuronal cell viability and ROS scavenging. Similarly, glucuronide conjugates also triggered the highest survival, attenuation of ROS levels, and inhibition of pro-inflammatory cytokines production in LPS-stimulated macrophages. These achievements highlighted the conjugated selectivity of dihydrocaffeic acid [3-(3′,4′-dihydroxyphenyl)propionic acid, DHCFA], of which the glucuronide form exhibited remarkable/potential neuroprotective and anti-inflammatory activity, whereas its sulfate form showed no obvious protective effects. It is important to note that most of these conjugated forms are rapidly metabolized and excreted, resulting in very low phenolic bioavailability. Therefore, the results reported in this paper can help further research to ascertain which mechanisms are responsible for the specific bioactivity of phenolic metabolites, and how microbial-derived phenolic acids and their conjugates affect potential hallmarks in the prevention of neurodegeneration disorders.

## Data Availability

The data presented in this study are available on request from the corresponding author. Data is not publicly available due to privacy.

## Abreviations

AD	Alzheimer’s disease
BBB	Blood–brain barrier
DCFDA	Dichlorodihydrofluorescein diacetate
DHPA	3,4-Dihydroxyphenylacetic acid
DHCFA	Dihydrocaffeic acid
DHCFAg	Dihydrocaffeic acid 3-O-β-D-glucuronide
DHCFAs	Dihydrocaffeic acid 3-O-sulfate
DPBS	Dulbecco’s phosphate buffered saline solution
IL-6	Interleukin-6
IL-8	Interleukin-8
LPS	Bacterial lipopolysaccharide
MTT	3-[4,5-Dimethylthiazol-2-yl]-2,5 diphenyl tetrazolium bromide
NALC	N-acetyl-L-cysteine
PCA	Protocatechuic acid
ROS	Reactive oxygen species
*t*BHP	*tert*-Butyl hydroperoxide
TNF-α	Necrosis factor alpha
